# Call for applications: Editor-in-Chief, *G3*

**DOI:** 10.1093/g3journal/jkab411

**Published:** 2022-01-04

The Genetics Society of America is seeking candidates for the position of Editor-in-Chief of *G3: Genes|Genomes|Genetics*. As the open access journal of the Society, *G3* provides a forum for the publication of high-quality foundational research—particularly research that generates useful genetic and genomic information.

The *G3* Editor-in-Chief (EIC), assisted by the Deputy Editor, leads an Editorial Board of seven Senior Editors and >100 Associate Editors who are all research-active scientists. The EIC works closely with Senior Editors to recruit and retain Associate Editors to cultivate an Editorial Board that represents a broad, deep, and diverse range of researchers and scientific expertise.

The EIC is responsible for fostering consistent decision-making across the journal, delivering clear, timely decisions and feedback to authors, and ensuring that the highest scientific and ethical standards are maintained. The EIC consults on submissions, collaborates with editors on decisions, and oversees consideration of authors’ appeals.

The EIC collaborates with Editorial Office staff (Executive Editor, Assistant Editor, Managing Editor), GSA staff, the Senior Editors, the Publications Committee, and the Editor-in-Chief of *GENETICS* to set journal strategy that supports GSA’s mission and core values.

The new EIC will build on existing strengths of the journal and identify opportunities for continuing development of the journal. The ideal candidate will have a vision to grow *G3*, including increasing its visibility in the international community and attracting new groups of authors, readers, and reviewers. The EIC will help identify trending and emerging topics for publication as new journal sections or in a themed series and continue to develop the journal as a valuable forum for the publication and discussion of important discoveries and ideas. The EIC will work toward a culture of inclusivity, keeping in mind the diverse audiences served by GSA and its journals, conferences, and programs.

## Terms of contract

The position officially begins January 1, 2023, but to provide for overlap with the current Editor-in-Chief, the appointee should start on or around October 1, 2022. The Editor-in-Chief serves a 5-year term, with the option to renew. GSA offers a stipend.

The EIC is expected to attend biannual meetings of the GSA Board of Directors, some GSA conferences, and publishing- or journal-related conferences (such as the National Academies Journal Summit or the Council of Science Editors Annual Meeting). In addition, the EIC travels at least once each year to meet with editorial staff, Senior Editors, and the GENETICS EIC. While it can vary, typically the EIC will serve 10–20 h per week.

The EIC of *G3* is a member of the GSA Board of Directors, reports directly to the Board, and is an *ex officio* member of the Publications Committee.

## Qualifications

Candidates should be established researchers with strong ties to the genetics and scientific community and a demonstrated track record of service, leadership, collaboration, and innovation. They should have a broad understanding and appreciation of topics in genetics and genomics, with a keen sense of emerging areas of inquiry. They should have an understanding of and empathy for the interests and needs of authors at all career stages.

At least 7 years of previous editorial experience, preferably at the Senior Editor level, is required. Candidates should have an interest in current topics relating to scholarly communications, such as publication ethics, public access mandates, open science, peer review, and reproducibility.

## About *G3*

*G3: Genes|Genomes|Genetics* is an open access journal providing a forum for the publication of high-quality foundational research—particularly research that generates useful genetic and genomic information such as genome maps, single-gene studies, genome-wide association and QTL studies, as well as genome reports, mutant screens, and advances in methods and technology. The Editorial Board of *G3* believes that rapid dissemination of these data is the necessary foundation for analysis that leads to mechanistic insights.

*G3* meets the critical and growing need of the genetics community for rapid review and publication of important results in all areas of genetics. *G3* offers the opportunity to publish the puzzling finding or to present unpublished results that may not have been submitted for review and publication due to a perceived lack of a potential high–impact finding. As part of our mission to serve our communities, we’ve published thematic collections that include Genomic Prediction, Fungal Genetics and Genomics, and Neurogenetics. *G3* has earned the DOAJ Seal, which is awarded by the Directory of Open Access Journals to journals that achieve a high level of openness, adhering to Best Practices and high publishing standards.

## About the Genetics Society of America

GSA serves an international community of scientists who use genetics to make new discoveries and improve lives. We advance biological research by supporting professional development of scientists, by communicating advances and fostering collaboration through scholarly publishing and conferences, and by advocating for science and for scientists. We seek to cultivate an inclusive, diverse research community that engages with the public, communicates the excitement and implications of discovery, and serves as an authoritative source of information.

See genetics-gsa.org for more information about GSA.

## Application deadline: May 1, 2022

Interested candidates should send a letter of intent and CV to Tracey DePellegrin, Executive Editor of *G3*, at g3_eic_search@thegsajournals.org.

